# Observations on the Frequency of the Occurrence of Follicular Ulceration in the Mucous Membrane of the Intestines, during the Course of Idiopathic Fever

**Published:** 1826-09

**Authors:** Cornwallis Hewett

**Affiliations:** Physician to St. George's Hospital.


### *Observations on the Frequency of the Occurrence of Follicular Ulceration in the Mucous Membrane of the Intestines, during the course of Idiopathic Fever.*

By Cornwallis Hewett, M.D. Physician to St. George's Hospital.

(Continued from page 114.)

The next point which I beg to propose for consideration, is the period of the fever at which these follicular ulcerations of the intestines first establish themselves, and are most likely to occur.

The history of the case of Charles Eynon shews that the first invasion of the fever took place about November 15th; that he was admitted into the hospital on the 22d November, and died on the 28th November, 1825.

Mary Hawkins, whose case has been already detailed, was admitted into the hospital on the evening of November 26th, 1825, and died during the course of the night, having been ill with fever following the suppression of the catamenia, from a cold caught at a dance only a week before: such, at least, was the history obtained through the kindness of the medical gentleman who had attended her previously to her admission into the hospital.

Another very rapidly fatal instance is that of the perforating ulcer, causing extensive peritonitis, in Sarah Rawden.

These instances, it must be acknowledged, far exceed the average rapidity of these follicular ulcerations in their progress to a fatal termination. But the possibility of the occurrence of follicular ulceration at so early a period of fever, appears to me to be a point of considerable practical importance. The fatal event may, indeed, be delayed from various causes: its most usual period is, probably, from the end of the second to the middle of the fourth week after the decided invasion of fever; but, previously to the establishment of the ulceration, it is obvious that some morbid action must have been going on in the parts, and the above investigations appear to have shown that the primary step of this morbid process is obstruction and enlargement of the mucous follicles; that inflammation, though not an unfrequent, is by no means a necessary, part of this process; that, even when ulceration is established, the exhalant surface in the immediate neighbourhood of such ulcers is sometimes found of its natural complexion, and without any increased vascularity whatever. It becomes, then, a very necessary point of enquiry to ascertain how soon, in the course of idiopathic fever, such enlargement of the mucous follicles has been detected; and here the researches of the French pathologists are calculated to afford us much assistance in the elucidation of this hitherto neglected subject.

In the Revue Médicale, Maii 1826, page 211, the case is related by Monsieur le Dr. Landini, of a young man, who had walked to the hospital on the 15th August, 1825, and was admitted with only the common symptoms "d'une dothin-entérite au huitième jour:" in the course of the night he had three evacuations, became delirious, and killed himself by throwing himself out of the window. In the post-mortem examination, the glandulæ aggregariæ, as well as the glandulæ solitariæ, were found considerably enlarged, and elevating the mucous membrane, without, however, any trace of erosion. They were particularly numerous and prominent in the ileo-cœcal portion of the intestines, "et faisaient proeminer dans le cœcum la valvule, qui avait l'apparence d'un bourrelet épais." It is remarkable, he adds, that the mucous surface immediately surrounding these little bodies, did not appear the least diseased.

In the examination of another case, where death had taken place on the sixth day after the invasion of the fever, the same appearances exhibited themselves, only with rather less development of the glands. The specimen was sent to M. Dupuytren, who exhibited it in the amphitheatre, and stated that he had, at another time, submitted a similar one to the inspection of the students.

In the Archives générales de Medicine, Janvier 1826, p. 70—76, M. Trousseau has detailed the daily change in the appearance of these glands, from the fifth day after the commencement of the fever until the fortieth. These observations were made after numerous dissections in the hospital at Tours, under the direction of M. Bretonneau, and are well deserving the attention of the profession; though the air of precision, with which the daily changes are announced, rather shakes than establishes one's faith.

It may suffice for our present purpose to state the result of his observations, that, unless the disease takes a favourable turn, and the enlarged glands begin to subside, slight erosion takes place at their summits between the tenth and twelfth days, and decided ulceration about the 14th; that, from the nineteenth to the twenty-first days, the ulcers, having become more superficial, tend to cicatrisation; that, by the twenty-fifth day, the glands have entirely subsided, and cicatrisation commences; that, by the twenty-fifth, it is generally completed, though still some ulcerations may occasionally be met with in the glands about the extremity of the ileum, but that the cases are rare where they are not all healed by the fortieth day.

That such is the series of changes produced in the condition of the mucous membrane of the intestines in these fevers, I am fully persuaded, though not quite prepared to fix their occurrence, and periods of succession, precisely to the dates lately mentioned.

The great difficulty, in a practical point of view, is to connect these morbid internal actions with the external symptoms. Until the follicles have attained sufficient size to irritate or disturb the mucous membrane in the exercise of its functions, the symptoms do not appear to exhibit any difference from those usually attending the common fever of this country. The only occurrence which, at this early period, would awaken my suspicions of the gradually advancing development of these mucous follicles of the intestines, is a peculiar hardness and fulness of the abdomen, not readily yielding to copious evacuations, whether spontaneously occurring or excited by purgative medicines. When, however, the mucous surface becomes irritated by the progressive enlargement of these follicles, then, in general, some obvious abdominal derangement, as painful, exhausting, and uncontrolable diarrhœa, accompanied with tension or obscure tenderness or nausea, on pressure of the abdomen, will generally supervene, and in its turn aggravate the other febrile symptoms. About or shortly after this period, apprehensions of some slight inflammatory affection of the intestines very probably may be excited, by the exasperation of the general as well as the local symptoms: or, very possibly, without any marked interval of excitement, the disease may gradually exhibit obscure, or even unequivocal, symptoms of ulceration of the intestines.

The detection of the existence of these follicular ulcers in the intestines, is by no means always so easy and simple a point as has been generally imagined: it might, indeed, *a priori*, have been expected that the occurrence of such ulcers must necessarily be attended with tormina, permanent pains and tenderness of the abdomen, with great irregularity of the bowels, and sanguineous, or at least morbid, evacuations; but a reference to even the few cases already detailed will show that these follicular ulcers have, on the post-mortem examination, been found to exist in the intestines, when the patient had been submitted daily to forcible pressure of the abdomen, without the acknowledgment of even any uneasiness,—when the abdomen has felt to the hand of the practitioner to be supple and natural,—when there has been little or no febrile heat of the skin,—when the pulse has been nearly natural,—when the tongue has appeared healthy,—when the evacuations have exhibited their natural appearance,—and when the patient, though subject to night-delirium, has recovered the possession of his faculties during the day, and retained them till the moment of his death.

I do not mean to say that any single case of fever has presented so favourable an assemblage of symptoms, and yet has exhibited, upon examination after death, the presence of follicular ulcers in the intestines; but that such ulcers have been found to exist, when most of the symptoms, usually deemed the requisite and infallible characteristics of their existence, have been absent: and again, in other cases, that no ulcerations have been detected after death in the intestines, notwithstanding the previous occurrence of the symptoms generally considered as tests of their existence.

It may, perhaps, be worth while briefly to investigate the value of some of those symptoms, the occurrence of which in the course of fever is usually thought to convey strong indications of the existence of intestinal ulcers; and, first, our attention will naturally be attracted to the consideration of intestinal hemorrhage.

That copious discharges of blood may take place from the intestines, or other mucous surfaces, without any permanent structural lesion, is a fact sufficiently known to every observant practitioner. Familiar examples occur in the frequency of hematemesis in the advanced stages of indurated liver, or when appearing as vicarious of the catamenial secretion; in menorrhagia also, and even in some cases of hæmoptysis, the same fact is exemplified. If the authority of great names is thought necessary for the establishment of this doctrine, it may suffice to allude to the observation of Boerhaave, that, when the refluent blood from the intestines is obstructed in its course through the vena portæ, it is apt to flow from the intestines. Morgagni made the experiment of tying the vena portæ in a living animal, and ascertained that it was speedily followed by an increased vascularity of the mucous membaane of the intestines, and sometimes by an exhalation of blood from its free surface. Bichat quaintly remarks, (vol. ii. p. 78,) "La matrice ne serait qu'un amas des cicatrices chez les femmes âgées, s'il y avait rupture dans la menstruation."

It seems desirable, then, to ascertain what means we have of distinguishing when the intestinal hemorrhage arises from a mere congestive state of the mucous membrane, and when from follicular ulcerations in it.

When a copious discharge of blood takes place from the intestines, without any breach of their mucous surface, in the course of fever, it usually occurs at rather an earlier period, and not unfrequently seems to act beneficially, by relieving the congestion of the abdominal viscera; but examples are not wanting where its occurrence, even at an advanced stage of fever, has gained the character, from the universal amendment accompanying it, of a critical discharge; and still, when the patient, after having become convalescent from the fever, has quickly sunk under the attack of some new malady, (as, for instance, of erysipelas,) no structural lesion has been discovered in the mucous membrane of the intestines in the post-mortem examination, though conducted with the greatest attention in order to ascertain this point, and though made within an interval of two or three weeks after the occurrence of such sanguineous evacuations.

The sanguineous evacuations, on the other hand, occasioned by follicular ulcers in the intestines, do not occur until a very advanced stage of the fever, and, when occurring, generally contain an enormous quantity of blood; the reason of which is sufficiently obvious, from the consideration that the hemorrhage does not take place until the ulcer has penetrated sufficiently deep to erode the ramifications of the mesenteric vessels: and, indeed, it is remarkable how frequently even their erosion is effected without the occurrence of hemorrhage. It is probable that, before their erosion is accomplished, they are often rendered impervious to the blood by the morbid actions accompanying the progress of the neighbouring ulceration, in a manner somewhat analogous to that by which the obliteration of the blood-vessels is often effected on the margin of vomicæ in the lungs, in phthisis tubercularis. When the intestinal hemorrhage is occasioned by follicular ulcers, the tenderness of the abdomen, which usually precedes the discharge of blood, does not seem to be diminished after it, but continues, at least for some days, equally perceptible as before the occurrence of hemorrhage; and the general symptoms, instead of improving, often become rapidly worse: whereas, when the hemorrhage arises without ulceration of the mucous surface, the relief thus afforded to the congestions in the abdominal viscera is followed by a relief also to the fulness and tenderness of the abdomen, and not unfrequently by an obvious general amendment.

But it frequently happens that the patient makes no complaint of pain in the bowels; that the appearance of the evacuations gives no indication of the existing ulcerations; and that their existence is first suspected, or detected, in consequence of the tenderness discovered on pressure of the abdomen; but, in using pressure with the hand as a means of ascertaining the existence of these intestinal ulcers, it is to be remembered that tenderness of the abdomen sometimes arises in fever from mere distention of the bladder, in consequence of the inability of the patient to void his urine; and that at other times it is only a part of the universal tenderness which so often affects the integuments in fever.

The right iliac region of the abdomen is the portion where the tenderness is most generally discovered, in consequence of the greater frequency of these ulcers in the neighbourhood of the ileo-cœcal valve; but, as the ulcers sometimes occupy so very small a space, it will be necessary that the whole of the abdomen be carefully pressed, in order that no ulcerated part may escape detection by pressure.

In some cases, at the time suspected, and afterwards proved, to be complicated with follicular ulceration of the intestines, though only a very slight degree of uneasiness was excited by forcible pressure upon the abdomen, still a very distressing degree of nausea resulted from it, and was most grievously complained of by the patient.

In estimating the degree of danger arising from these intestinal ulcers in fever, it may be useful to remember that not only a large extent of confluent, though superficial, ulcers, but even a single deeply-penetrating ulcer, will be attended with considerable danger, increasing in proportion to its approximation to the peritoneum, and the risk of its causing hemorrhage, by erosion of some mesenteric vessel, or of its exciting extensive inflammation of that membrane, whether before or after perforation of it. The more exquisite tenderness of the abdomen, with the supervention of deadly sickness, and the more unequivocal symptoms of peritonitis, will generally give sufficiently clear indications of this extension of the ulceration.

TREATMENT.

After these desultory remarks upon the symptoms usually attending and indicating the existence of follicular ulcerations in the intestines in fever, I beg, with every proper feeling of deference, to submit the following outline of a plan of treatment, as calculated, if acted upon at a sufficiently early period, to prevent their occurrence ; or, if adopted after the establishment of the ulcerations, to conduct them to a favourable termination.

The French practitioners seem to be labouring under an impression that it is no more within the power of medical science to control the appearance or disappearance of this follicular affection, than of measles, variola, scarlatina, or other specific inflammations ;—(qu'on ne peut pas plus empêcher l'apparition ou hâter la disparition de la dothinentérite, qu'on n'empêche ou qu'on ne hâte celle de la rougeole, de la variole, de la scarlatine, et autres inflammations spécifiques," &c.)*—but, though I most willingly admit the excellence of their pathological researches, I feel myself compelled to state my dissent from the above doctrine, and its accompanying principles of practice.

In the proposed plan of treatment, the indications will necessarily vary according to the stage of the disease, but will be principally derived from the supposed progressive alteration in the condition of the follicular glands of the intestines.

There appear to be three stages of the disease sufficiently distinct to be recognised by the external symptoms, and requiring a corresponding variety of treatment.

The first stage comprises the period between the incipient and the complete development of the mucous follicles, which is prior to any inflammation or erosion of the neighbouring membrane.

The second comprehends the period of the incipient and advancing ulceration of the mucous follicles, which is generally, but not invariably, accompanied with some slight inflammation of the neighbouring exhalant surface.

The third embraces the period of the separation, by sloughing, of these previously disorganised follicles, with their neighbouring structures; and the termination of the ulcers, either in cicatrisation or in the death of the patient.

In conformity with the pathological views which have been already detailed, the plan of treatment proposed for the first stage of fever consists in the effective exhibition of Calomel,

* Revue Medicale, Maii 1826; p. 204.

combined with any of the auxiliary purgatives and sudorifics; the doses of each being regulated according to the strength and age of the patient.

In addition to the indications derived from the inspection of the evacuations, and the degree of improvement of the general febrile symptoms, a practically useful hint for the continuance or discontinuance of the purgatives may be obtained by feeling the abdomen of the patient. After three or four days of this treatment, it will probably have become less hard and prominent than it at first appeared to be; but, until the abdomen feels soft and supple (as in its healthy state), purgation, though not so actively, is still to be daily pursued.

When this system has been followed for five or six days, in the first stage of fever, if the evacuations still exhibit a morbid appearance, and the abdomen, though not so hard and resisting as at first, still remains tumid and tympanitic, it is sometimes of service to suspend for a day or two the use of the purgatives, and exhibit a small dose daily (two or three drachms) of castor-oil, for the purpose of ascertaining whether the unhealthy character of the evacuations may not have been prolonged by the continued irritation of the purgatives. Laxative doses of castor-oil, thus administered, will often produce, in the course of twenty-four or thirty-six hours, well-coloured though liquid stools, and an accompanying subsidence of the abdomen to its naturally soft and supple condition.

But if, after this intermission of the purgatives, such an amendment is not discovered in the condition of the abdomen, and in the appearance of the evacuations, it will be advisable to resume the use of the cathartics, in milder doses.

It may be objected, that this system of purging freely is inadmissible in many cases of fever; in such, for instance, as are attended with early diarrhœa: but these early watery evacuations appear to be principally derived from the exhalant surface, and do not seem by any means to be incompatible with a torpid condition of the mucous follicles of the intestines. At any rate, purgative doses of Calomel and Rhubarb, or of Blue-pill and Rhubarb, or of Hydrargyrum cum Cretâ and Rhubarb, or of Hydrargyrum cum Cretâ and Ipecacuanha, are often found to be most effectual in controlling them, and in reducing the tension of the abdomen which usually accompanies them.

In addition to the many other benefits derived from purgatives at this early stage of fever, they appear to me to be particularly effective in preventing follicular ulceration of the intestines, by the power which they possess of clearing out the obstructed orifices, and emptying the distended follicles of their morbidly-inspissated secretions; so that these mucous glands, thus seasonably relieved, soon subside, and resume their natural size and healthy functions.

The treatment of the second stage requires to be regulated upon the principle of subduing the inflammatory action, if the attendant symptoms give reason to apprehend that any such exists, of the membrane immediately adjoining the enlarged follicles, and of restoring these mucous glands to their natural size, and the exercise of their proper functions.

These indications seem to be best fulfilled by the repeated application of leeches to the abdomen, more or less frequently according to the degree of its tenderness, intensity of the fever, and strength of the patient; while two or three grains of Calomel, and the same quantity of James's powder, may be given in pills, and repeated every six, eight, or twelve hours; with the occasional exhibition, if the bowels are not sufficiently soluble, of small doses of Castor-oil; or, if they be too much relaxed, three grains of the Extract of Poppies, or a quarter of a grain of Opium, may be added to each dose of the above pills; or, in lieu of them, Hydrargyri cum Cretâ ℈ss. may be given, and repeated three or four times daily. Hot fomentations are at the same time to be kept constantly applied to the abdomen; and afterwards they may be superseded by the application of a blister.

The same measures may be continued even after the follicles have passed into a state of ulceration, provided that the symptoms indicate a continuance of the local inflammation and febrile excitement.

It may fairly be asked, why venesection has not been decidedly recommended in the second stage of this disease? In some cases it is undoubtedly advantageous, but only in very few; for, previously to the establishment of any inflammatory action, and still more so to the exhibition of any symptoms indicating its existence, so much structural lesion has generally been effected in the follicular glands and neighbouring structures, as to render the patient incapable of receiving benefit from venesection, or even of bearing it, if pushed to any extent.

The insidious progress of these follicular ulcers, until the moment of their exciting peritonitis, and the inability of the patient to bear the degree of depletion necessary for its cure, are well exemplified in the fifth Case. This patient was upon the point of recovering from an attack of fever, when she suddenly experienced a relapse. On the Friday morning (seven o'clock A.M.) she had been bled almost to syncope, for unequivocal symptoms of peritonitis. When I first saw her, (at ten o'clock A.M.) I immediately directed a repetition of the venesection, and repeated it again in the afternoon; and followed it up by the application of leeches in the evening. Venesection was repeated, for the fourth time, at two o'clock P.M. on the Saturday. The venesection, on all these occasions, was pushed almost to syncope; but only a temporary mitigation of the sufferings was obtained after each venesection.

At eleven o'clock P.M. she was incessantly complaining both of the pain of the blister and also of the internal pain, which she compared, in her own emphatic language, to "a cauldron of pitch and sulphur burning within her;" the skin was quite cold and clammy; and she expired on the following morning at eight o'clock.

The dissection showed that the measures employed had subdued the peritoneal inflammation, but could not counteract the structural lesion previously effected by two small but deep follicular ulcers, discovered within an inch of the ileocœcal valve.*

In the next case, the coldness of the skin, the softness and weakness of the pulse, and the other symptoms of excessive debility, were sufficiently obvious to convince all, who saw the case at the time of its admission, that venesection and depletory measures were then perfectly inadmissible. She was admitted into the hospital about two o'clock P.M. May 24th, and died on the afternoon of May 26th.

The dissection clearly proved that the peritonitis was not the primary disease, but had been excited by the progress of one of the follicular ulcerations, which had penetrated through all the coats of the ileum.

In the history of seven fatal cases of similar perforations of the small intestines by the progress of ulcers from the mucous membrane, by M. Louis, it is remarked, "the interval of time elapsing between the first symptoms of perforation and death has varied from twenty to forty-eight hours." This author also states, that the disease came on, in all these cases, as a slight continued fever, scarcely presenting any severe symptom till the period of the perforation. Diarrhœa was constant

* Vide page 102 of the last Number of this Journal.

and severe in one patient only; it was moderate in another; present, but still more inconsiderable, in a third; and in the four others it was altogether absent. The pain in the belly was slight, and experienced only at intervals; while the patient who had the severe diarrhœa had no pain at all. Four regarded themselves as convalescent, and had been considered as such for several days, when the symptoms of perforation supervened; so that no unfavourable occurrence could reasonably have been anticipated. The treatment was nearly the same in all: bleeding, both general and local, was had recourse to, without the slightest apparent influence over the disease. The antiphlogistic plan was vigorously employed in the second case,—(" la methode antiphlogistique n'a certainement pas été employée avec trop de reserve chez le second;")—yet it proved one of the most speedily fatal.

When, however, the third stage has arrived,—viz. that of the separation, by slough, of the previously disorganised follicles,—the accomplishment of this process, and the subsequent reparation of the ulcerated surfaces, appear to be most effectually promoted by the same constitutional tonic treatment as that by which the cure is best effected of the sloughing sores so often found, at this period of fever, on the outer surface of the body; great attention being paid to keep the bowels in a gently relaxed condition, by the mildest possible means, as very small doses of castor-oil or slightly-laxative enemata.

The above hints have been very humbly suggested, with the view of pointing out a line of treatment adapted to the relief of those cases only in which abdominal derangements or lesions are the prominent feature of the fever.*

* Of the ten cases of Follicular Ulcerations related in the former part of this paper, four were under the care of Dr. Hewett, and the remaining six under that of the other physicians of St. George's Hospital.

