# Erratum

**Published:** 1826-10

**Authors:** 


*Erratum.*—In our last Number, page 248, line 30 from the top, *dele* the word " without."

